# Blood transfusion after cardiac surgery increases the hospital length of stay in adult patients

**DOI:** 10.1186/cc11054

**Published:** 2012-03-20

**Authors:** L Hajjar, JL Vincent, J Almeida, F Jatene, A Rodrigues, J Fukushima, R Nakamura, C Silva, E Osawa, R Kalil, F Galas, J Auler

**Affiliations:** 1 Heart Institute, São Paulo, Brazil; 2 Erasme Hospital, Université libre de Bruxelles, Belgium

## Introduction

Transfusion of allogeneic red blood cells (RBC) is a recognized risk factor for adverse outcomes following cardiac surgery. A potential endpoint to assess clinical complications and incremental use of resources is the measurement of hospital length of stay (LOS). The primary objective of this study was to evaluate the relationship between blood transfusion and increased hospital LOS after cardiac surgery.

## Methods

A prospective observational substudy that analyzed data from the overall 502 patients enrolled in the Transfusion Requirements After Cardiac Surgery (TRACS) study [1]. Patients who received blood transfusion during surgery or ICU stay were further categorized according to the number of prescribed RBC units: nontransfusion group, low transfusion requirement group (3 units or less), and high transfusion requirement group (more than 4 units).

## Results

Patients who received any RBC unit had longer median LOS than patients in the nontransfusion group: 15 days (95% CI, 12.66 to 17.34) in high transfusion requirement group versus 10 days (95% CI, 9.1 to 10.9) in low transfusion group versus 8 days (95% CI, 7.4 to 8.6) in nontransfusion group (*P *< 0.001). In a multivariate Cox proportional hazards model the following factors were considered predictive: age older than 65 years (hazard ratio (HR), 1.38 (95% CI, 1.11 to 1.73); *P *= 0.004), EuroSCORE 3 to 5 (HR, 1.44 (95% CI, 1.12 to 1.86); *P *= 0.005), EuroSCORE higher than 5 (HR, 1.7 (95% CI, 1.26 to 2.28); *P *< 0.001), valvular surgery (HR, 1.57 (95% CI, 1.26 to 1.95); *P *< 0.001), combined procedure (HR, 1.6 (95% CI, 1.03 to 2.46); *P *= 0.034), bypass duration higher than 100 minutes (HR, 1.23 (95% CI, 1.01 to 1.51); *P *= 0.046), LVEF lower than 40% (HR, 1.69 (95% CI, 1.24 to 2.32); *P *= 0.001), LVEF 40 to 59% (HR, 1.36 (95% CI, 1.1 to 1.69); *P *= 0.004), RBC transfusion of 1 to 3 units (HR, 1.24 (95% CI, 1.01 to 1.53); *P *< 0.001), and RBC transfusion >3 units (HR, 1.96 (95% CI, 1.45 to 2.66); *P *< 0.001). In an adjusted model for age, EuroSCORE, type of surgical procedure, LVEF and cardiopulmonary bypass time, the exposure to RBC transfusion was associated with an elevated LOS.

## Conclusion

Blood transfusion is an independent risk factor for prolonged hospital LOS after cardiac surgery. This finding can support the development of blood conservation strategies in order to avoid deleterious outcomes of blood exposure.
